# The effectiveness of teach-back health education combined with high-quality nursing in patients with atrial fibrillation receiving anticoagulant treatment

**DOI:** 10.12669/pjms.41.4.11676

**Published:** 2025-04

**Authors:** Chunhui Zhao, Zhao Zhao, Linghui Song

**Affiliations:** 1 Chunhui Zhao Department of Cardiology I, Zhangjiakou First Hospital, Zhangjiakou City, Hebei Province, 075000, China; 2 Zhao Zhao Department of Cardiology I, Zhangjiakou First Hospital, Zhangjiakou City, Hebei Province, 075000, China; 3 Linghui Song Department of Cardiology I, Zhangjiakou First Hospital, Zhangjiakou City, Hebei Province, 075000, China

**Keywords:** Anticoagulation, Atrial fibrillation, High-quality nursing, Teach-back health education

## Abstract

**Objective:**

To explore the efficacy of combining teach-back health education and high-quality nursing in the anticoagulation treatment of patients with atrial fibrillation (AF).

**Methods:**

Clinical data of 110 patients with AF admitted to the Zhangjiakou First Hospital between June 2021 to August 2023 who received anticoagulation therapy were retrospectively analyzed. Among them, 53 patients received routine high-quality nursing care (control group), and 57 patients received teach-back health education combined with high-quality nursing (observation group). Exercise of Self-Care Agency (ESCA) scale scores, treatment adherence, mastery of health knowledge, and nursing satisfaction before and after intervention were assessed.

**Results:**

After the intervention, the scores of self-care skills, self-responsibility, self-concept, and health knowledge in both groups significantly increased compared to pre-intervention and were significantly higher in the observation group compared to the control group (P<0.05). The post-intervention adherence rate of the observation group (96.49%) was higher than that of the control group (84.91%) (P<0.05). Similarly, the mastery of health knowledge and nursing satisfaction in the observation group were significantly higher compared to the control group (94.74% and 92.98% versus 81.13% and 79.25%, respectively; P<0.05).

**Conclusions:**

Implementing teach-back health education combined with high-quality nursing during anticoagulation treatment for patients with AF can deepen the patient’s understanding of disease and health knowledge, improve self-care ability and treatment adherence, and increase satisfaction.

## INTRODUCTION

Atrial fibrillation (AF) is the most common form of cardiac arrhythmia^1^ that may lead to complications such as heart failure, and thromboembolism, if left untreated.^1^,^2^ In recent years, with the gradual aging of the general population, the incidence rate of AF has been on the rise.^3^ AF can affect the patient’s quality of life, exercise endurance, and hemodynamic fluctuations and is associated with a significant socioeconomic burden.^3^–^5^ Anticoagulant therapy is an important intervention for patients with AF.^6^ However, due to the patient’s lack of correct understanding of the disease and poor treatment adherence, treatment efficacy and overall prognosis are often not satisfactory.^6^,^7^ Therefore, effective nursing interventions are essential for improving the effectiveness of anticoagulation therapy for AF.

Studies have shown that structured, high-quality nursing care of patients with AF was associated with considerable improvement in patients’ adherence to therapies and, subsequently, lower cardiovascular mortality and hospitalizations.^8^–^11^ In addition to nursing care, health education is crucial for improving patients’ engagement and adherence to the treatment process and treatment plans.^12^,^13^ The teach-back method of patient education, a technique of verifying patients’ understanding of their health information, has been demonstrated as beneficial in reinforcing patient education.^12^ However, the teach-back method has not yet been fully studied in patients with AF.

This study aimed to clarify the effectiveness of the teach-back health education method combined with high-quality nursing in patients with AF who receive anticoagulation therapy. Our results may provide a practical reference for the nursing management of AF.

## METHODS

Clinical data of 110 patients with AF (66 males and 44 females) who received anticoagulation therapy in Zhangjiakou First Hospital from June 2021 to August 2023 were retrospectively selected. Patients’ ages ranged between 47 and 74 years, with a median age of 62 (55-67) years; 53 patients received high-quality nursing care and were set as the control group; 57 patients received teach-back health education combined with high-quality nursing and were designated as the observation group.

### Ethics Statement:

All procedures performed in this study involving human participants were in accordance with the ethical standards of the institutional and/or national research committee, and the 1964 Declaration of Helsinki and its later amendments or comparable ethical standards. The study was approved by the Ethics Review Board of Zhangjiakou First Hospital (No. 2023L0221; Date: 23-September, 2023).

### Inclusion criteria:

- Patients diagnosed with AF through electrocardiogram examination and clinical symptom evaluation.^14^
- Received anticoagulant treatment.
- Complete clinical data.

### Exclusion criteria:

- Patients with secondary AF caused by heart valve disease, hyperthyroidism, or cardiac surgery.
- Patients with malignant tumors.
- Patients with end-stage diseases.
- Patients with mental illness.
- Patients with obvious active bleeding and coagulation dysfunction.
- Patients with severe organic lesions in other organs.
- Patients with speech communication barriers.

### High-quality nursing care:

### Enhanced communication with patients:

Nursing personnel collected the patient’s history of drug allergies, family history, and dietary habits. The patients were educated with detailed information about the disease, risk factors, and related precautions through oral communication.

### Medication care:

Since there is a risk of bleeding during anticoagulant therapy, and there are significant individual differences in the dosage of medication (if the patient has liver and kidney dysfunction, the dosage of medication should be appropriately reduced, and adverse drug reactions should be closely monitored), a history of bleeding was clarified, and platelet and coagulation function of each patient was checked before beginning anticoagulant therapy. Patients were advised to follow the prescribed medication regimen strictly, and their international normalized ratio (INR) was regularly reviewed. The dosage of medication was adjusted promptly based on the INR test results. Patients were closely monitored for any adverse reactions during the course of the therapy, such as pruritis, vomiting, and nausea.

### Bleeding management:

Strict bleeding monitoring was enforced during anticoagulant therapy, and included regular evaluation of possible intestinal bleeding, subcutaneous ecchymosis, gingival bleeding, purpura, and hematemesis. In case of abnormal bleeding was detected, the physician was immediately informed to implement the targeted intervention.

### Dietary guidance:

A dietary plan was developed based on the patient’s preferences, disease characteristics, and physical condition. Patients were advised to consume more nutritious and easily digestible foods, reduce their vitamin K intake, and avoid alcohol.

Patients were encouraged to maintain a regular lifestyle, minimize emotional fluctuations, and maintain smooth and regular bowel movements.

### Psychological care:

The psychological characteristics of patients were assessed through conversation, providing corresponding interventions to alleviate or eliminate negative emotions related to depression and anxiety, patiently addressing any arising doubts, and assisting the patients in maintaining an optimistic attitude.

### Teach-back health education:

All providers received one-month standardized and professional training before implementation.

### Information transmission:

Corresponding methods were selected to explain the basic characteristics of AF, relevant precautions, anticoagulant treatment principles, and common adverse reactions during disease treatment. Appropriate methods were chosen based on the patient’s general condition, educational level, and family support level and included pictures, PowerPoint presentations, and other means. Disease-related health knowledge was transferred through social applications and other means, and a communication platform was established. Patients were encouraged and guided to openly and actively express their rehabilitation experiences. Special personnel were available on communication platforms to promptly answer patients’ questions during the treatment and provide professional answers to personalized questions.

### Effect evaluation:

A comprehensive and systematic evaluation of the patient’s self-management behavior, rehabilitation training knowledge, and psychological state was done. Patients were guided to express themselves through any means necessary. If required, the health education content was repeated using body movements and other forms of communication.

### Clarification:

Nursing personnel promptly clarified and corrected inappropriate or omitted content in the patient’s retelling and repeated health education when required. If necessary, health education and communication with patients employed phrases such as “Maybe my expression is not very easy to understand, I will explain it to you again in another form” based on their specific conditions.

### Understanding:

Open-ended questions, such as “What are the common self-management behaviors for AF,” “What are the purposes and mechanisms of anticoagulation therapy for AF?” and “What are the routine rehabilitation training measures for AF? “ were used. Health education was considered completed if the patient was able to provide accurate and complete answers. In all other cases, the above content was repeated.

### Observation indicators:

### Self-care ability before and after intervention:

The Exercise of Self-Care Agency (ESCA) scale was used. The ESCA scale involves four dimensions, namely self-care skills (12 items, 48 points in total), self-responsibility (8 items, 32 points in total), self-concept (9 items, 36 points in total), and health knowledge level (14 items, 56 points in total), with a total of 43 items. Each item scores 0-4 points, and the score is positively correlated with the patient’s self-care ability. The Cronbach’s scale coefficient is 0.874.

### Treatment adherence:

Simplified Medication Adherence Questionnaire (SMAQ) was adopted. It consists of six questions, each with a “yes” or “no” answer, where “yes” is one point and “no” is zero points; The total score is six points, a total score of zero indicates good adherence, 1-2 indicates moderate adherence, and ≥ three indicates poor adherence.

### Health knowledge:

A self-designed scale was used for the self-assessment. The assessment included the pathogenesis of AF, daily precautions, dietary knowledge, anticoagulant treatment knowledge, etc., with a total score of 10 points. Based on the score, health knowledge of patients was classified as good (9-10 points), moderate (6-8 points), and poor (≤ 5 points).

### Nursing satisfaction:

The Newcastle Satisfaction with Nursing Scale (NSNS) was used for assessment: very satisfied (95 points), satisfied (76-94 points), generally satisfied (57-75 points), dissatisfied (38-56 points), very dissatisfied (19-37 points). Very satisfied, satisfied, and generally satisfied were included in the total satisfaction.

### Statistical analysis:

All data analyses were conducted using SPSS software (version 20.0; IBM Corp, Armonk, NY, USA) and PRISM 8.0 software (GraphPad, San Diego, USA). The Shapiro–Wilk test was used to evaluate the normality of the data. The data with normal distribution were represented by mean ± standard deviation, an independent sample t-test was used for inter-group comparison, and a paired t-test was used for intragroup before and after comparison. The non-normally distributed data were represented by median and interquartile intervals and were analyzed using Wilcoxon’s test. Data were counted using the Chi-square test to represent the number of use cases. *P*<0.05 was statistically significant.

## RESULTS

**Fig.1:**
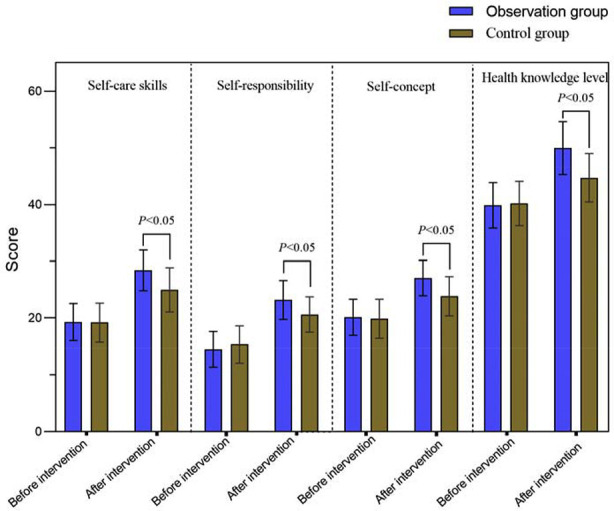
Comparison of self-care abilities between two groups.

A total of 110 patients met the study criteria. Among them, 53 patients in the control group received high-quality nursing care, whereas the observation group of 57 cases received a combination of teach-back health education and high-quality nursing. There was no significant difference in the baseline data between the two groups (*P*>0.05) (Table-I). Before the intervention, the two groups had no significant differences in self-care skills, self-responsibility, self-concept, and health knowledge level scores (*P*>0.05). After the intervention, the self-care skills, self-responsibility, self-concept, and health knowledge level scores of the two groups increased compared to pre-intervention and were significantly higher in the observation group than in the control group (*P*<0.05) (Fig.1).

**Table-I:** Comparison of baseline data between two groups.

Group	Gender (male/female)	Age (years)	Disease course (year)	Type of AF	
				Paroxysmal AF	Persistent AF
Observation group (n=57)	33/24	62(54-67)	2(2-3)	20 (35.09)	37 (64.91)
Control group (n=53)	33/30	63(58-68)	2(2-3)	17 (32.08)	36 (67.92)
*χ^2^/t/Z*	0.218	-0.671	-0.955	0.112	
*P*	0.640	-0.502	0.339	0.738	

**Fig.2:**
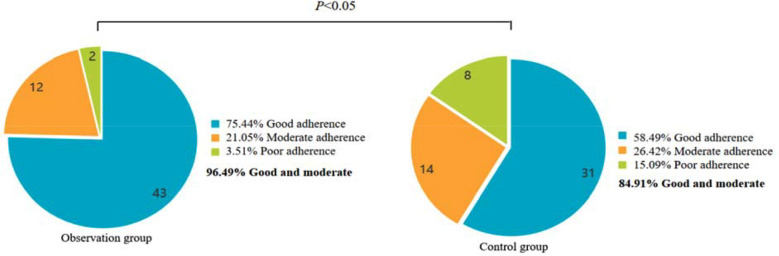
Comparison of treatment adherence between two groups.

**Fig.3:**
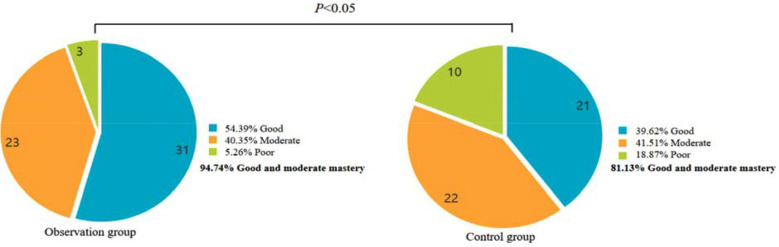
Comparison mastery of health knowledge between two groups.

**Fig.4:**
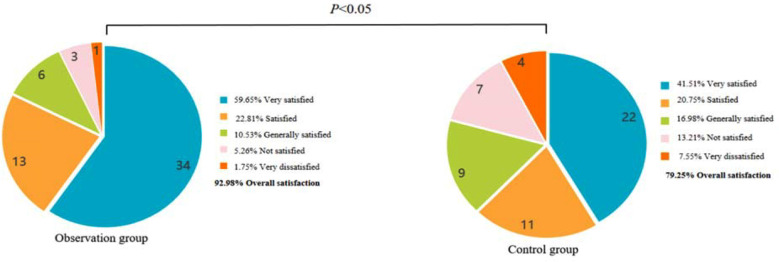
Comparison of nursing satisfaction between two groups.

The adherence rate of the observation group (96.49%) was significantly higher than that of the control group (84.91%) (*P*<0.05) (Fig.2). The excellent health knowledge in the observation group (94.74%) was significantly higher than that in the control group (81.13%) (*P*<0.05) (Fig.3). Nursing satisfaction in the observation group (92.98%) was significantly higher than in the control group (79.25%) (*P*<0.05) (Fig.4).

## DISCUSSION

This study showed that implementing teach-back health education combined with high-quality nursing during anticoagulation treatment for patients with AF is associated with higher ESCA-related dimension scores and improved the adherence and the mastery of health knowledge compared to high-quality nursing alone. Our results suggest that in AF patients undergoing anticoagulation therapy, teach-back health education should be implemented together with high-quality care, as it helps enhance patients’ understanding of the disease, thereby ensuring treatment adherence rate and encouraging active cooperation with treatment and care.

Cognitive-behavioral theory suggests that cognitive behavioral therapy leads to significant improvement in functioning and quality of life.^15^ Since teach-back health education focuses on maintaining a respectful and caring attitude during the intervention period, the intervention content and form are straightforward and easy to understand. A patient’s level of mastery of health knowledge can affect their behavioral changes, thereby improving their self-care ability and treatment adherence rate. In agreement with our results, previous research pointed out that special attention should be paid to guiding patients to retell the health education content using their own forms of expression^16^ not only does it help educators fully assess the patient’s understanding and mastery of health knowledge, but further intervention can also be provided to address patient omissions and misunderstandings, ensuring that patients truly understand and master health content. Such an approach ensures that the patients know the benefits of maintaining healthy behavior in ensuring good disease outcomes, encouraging them to cooperate actively with treatment and care, thus improving the effectiveness of treatment and the disease prognosis.^16^,^17^

There are relatively few clinical reports and studies on the application value of teach-back health education in AF. However, previous studies have confirmed the application value of this health education model in other diseases.^16^,^18^-^22^ A systematic review by Oh et al.^20^ showed that implementing the teach-back health education model can effectively reduce the 30-day readmission rate. The review emphasized that strict requirements for the educational methods and attitude of medical staff that the teach-back health education model enforces, its focus on health education and nursing interventions that aim to ensure a caring and friendly environment were all instrumental in alleviating patients’ anxiety, ensuring the quality and effectiveness of health education, and enhancing patient adherence. 20 The US Healthcare Research and Quality Agency promoted the teach-back health education model as one of the most effective comprehensive preventive measures that not only deepen patients’ understanding of the causes, risk factors, diagnosis, treatment, protective and rehabilitation measures, and disease prognosis but also improve patient treatment adherence. 21

Moreover, since the teach-back health education model is a two-way transmission model between nurses and patients, its health education effect is not only influenced by the guiding role of health educators but also by patient-related factors. Educators can thus refer to the patient’s restatement of the effectiveness of health education content to ensure intervention effectiveness.^21^ The research results of Oh et al.^16^ showed that implementing a teach-back health education model for discharged patients with heart failure was associated with improved self-care management, symptom perception, and self-care maintenance abilities. The research results of Mashhadi et al.^22^ indicate that implementing teach-back health education for discharged patients through mobile medical platforms can significantly reduce readmission rates and ensure a good prognosis for the disease.

The results of this study also showed that combining the teach-back health education model with routine nursing was associated with considerably increased nursing satisfaction compared to routine nursing alone (*P*<0.05). Our results are consistent with the previous research. Hodges et al.^23^ found that teach-back health education can improve the satisfaction of nursing care for discharged patients in the emergency department. Implementing the model resulted in an improved understanding of the disease and the rehabilitation process. It was able to alleviate depression and anxiety caused by a lack of disease cognition and improve patients’ self-care ability and initiative. Patients could effectively and actively participate in disease treatment and rehabilitation, which ensured the effectiveness of disease intervention and increased overall patient satisfaction. Similar conclusions were reported by Marks et al.^24^ Improved rehabilitation and patient satisfaction, may also reduce social and economic burden as it can help to avoid costly hospitalization, reduce hospital length of stay, and prevent re-admissions. Therefore, the results of this study may not only provide a practical reference for the nursing management of AF, but also provide evidence of its cost benefits for both individuals and society.

### Limitations:

This study has some limitations. First, due to being a single center study, there may be certain regional and cultural limitations, which may result in insufficient representativeness of the sample, and the results may not be fully generalizable to other regions or populations. Second, which components of the teach-back method had the greatest impact was not studied, which could be further studied in future research. Third, the potential reduction in stroke risk or expected stroke rate that was a direct result of this combined intervention was not studied. Fourth, long-term clinical efficacy could not be achieved without a long-term follow-up. Finally, the impact of the two nursing methods on the long-term functional recovery of patients was not analyzed. Further high-quality research is needed to verify our conclusions.

## CONCLUSION

Implementing the teach-back health education model combined with high-quality nursing during the anticoagulation treatment for AF patients can deepen their understanding of disease and health knowledge, improve self-care ability and treatment adherence, and increase overall satisfaction.

### Authors’ Contributions:

**CZ:** Study design, literature search, manuscript revision, validation, critical analysis and manuscript writing

**ZZ: and LS:** Data collection, data analysis and interpretation. Critical review.

All authors have read, approved the final manuscript and are accountable for the integrity of the study.
